# A Panel of Broad-Spectrum Antivirals in Topical Ophthalmic Medications from the Drug Repurposing Approach during and after the Coronavirus Disease 2019 Era

**DOI:** 10.3390/jcm9082441

**Published:** 2020-07-30

**Authors:** Pietro Emanuele Napoli, Lorenzo Mangoni, Pietro Gentile, Mirco Braghiroli, Maurizio Fossarello

**Affiliations:** 1 Clinica Oculistica, San Giovanni di Dio Hospital, Azienda Ospedaliera Universitaria di Cagliari, 09124 Cagliari, Italy; lollomangoni92@gmail.com (L.M.); gentilepietro92@gmail.com (P.G.); mirco.mbmb@gmail.com (M.B.); maurizio.fossarello@gmail.com (M.F.); 2 Department of Surgical Sciences, Eye Clinic, University of Cagliari, 09124 Cagliari, Italy

**Keywords:** coronavirus disease 2019 (COVID-19), coronavirus, severe acute respiratory syndrome coronavirus 2 (SARS-CoV-2), eye drop, therapy, treatment, antiviral, repurposing, drug, ocular surface

## Abstract

The coronavirus disease 2019 (COVID-19) represents a global concern of public health caused by severe acute respiratory syndrome coronavirus 2 (SARS-CoV-2). Its clinical manifestations are characterized by a heterogeneous group of symptoms and pictures (ranging from asymptomatic to lethal courses). The prevalence of conjunctivitis in patients with COVID-19 is at present controversial. Although it has been reported that only 0.9% developed signs of conjunctivitis, other report indicates that up to 31.6% of hospitalized patients had conjunctivitis. Considering the widespread use of topical ophthalmic medications (e.g., eye drops) by the general population, for various reasons (e.g., artificial tears, anti-glaucoma medications, topical antibiotics, etc.), the existence of their side effects as antiviral action should be investigated in-depth because it could possibly explain the aforementioned controversial data and represent a potential antiviral treatment for SARS-CoV-2 replication/diffusion on the ocular surface. Here, we discuss and elucidate the antiviral side effect of many eye drops and ophthalmic ointments commonly used for others purposes, thus showing that these secondary effects (not to be confused with the ‘adverse effects’) might be of primary importance in a number of viral infections (e.g., those for which there is no validated treatment protocol), according to a drug repurposing approach. Some active ingredients or excipients described here have activity against other types of viruses, thus suggesting potential broad-spectrum applications.

## 1. Introduction

The coronavirus disease 2019 (COVID-19) represents a global concern of public health that quickly spread around the world in early 2020 [1,2]. The origin of this disease is infectious, being caused by severe acute respiratory syndrome coronavirus 2 (SARS-CoV-2), and its clinical manifestations are characterized by a heterogeneous group of symptoms and pictures (ranging from asymptomatic to lethal courses).

Ocular involvement of COVID-19 and the potential role of the eye as a transmission route of SARS-CoV-2 have been previously described by several authors [3]. Accordingly, the detection of specific cell-surface receptors on the ocular surface, called angiotensin converting enzyme 2 (ACE2), and proteins promoting the binding between the virus and the host cell, named transmembrane protease serine 2 (TMPRSS2), is of considerable importance for understanding the presence of SARS-CoV-2 at eye level [3].

Apart from the problem of interpersonal transmission of COVID-19 through conjunctiva and the relative issues on the presence and replication of the virus at the level of human tears and ocular surface epithelia, the appearance of conjunctivitis in COVID-19 may be presumed to be not uncommon on the basis of inherent ocular tropism of other viral upper respiratory tract infections (adenovirus being the most common). Although the eyes are not the main transmission routes of SARS-CoV-2 [4], conjunctivitis can be the first presenting symptom of COVID-19, before the appearance of other symptoms, such as cough and fever [5,6,7].

The prevalence of conjunctivitis in patients with COVID-19 is, at present, controversial. Although it has been reported that only 0.9% of patients developed signs of conjunctivitis [8], another report indicates that up to 31.6% (12 cases out of 32 patients) of hospitalized patients had conjunctivitis [9].

Considering the widespread use of topical ophthalmic medications by the general population for various reasons (e.g., artificial tears, anti-glaucoma medications, topical antibiotics, etc.), the existence of side effects as an antiviral action should be investigated in-depth because it could possibly explain the aforementioned controversial data and represent a potential antiviral treatment for SARS-CoV-2 replication/diffusion on the ocular surface.

Here, we discuss and elucidate the antiviral side effect of many eye drops and ophthalmic ointments commonly used for others purposes, thus showing that these secondary effects (not to be confused with the “adverse effects”) might be of primary importance in a number of viral infections (in particular, those for which there is no validated treatment protocol), according to a drug repurposing approach.

## 2. Drug Repurposing Approach: Potential Antiviral Action of Drugs in Eye Drops and Ophthalmic Ointments

An attractive alternative strategy of drug discovery is that of the drug repurposing approach, which eliminates the high costs and the time required for the *de novo* drug development. This is possible through the identification (sometimes accidental or fortunate) of some side effects (which are different from the adverse effects) of existing and available (approved) drugs. A famous example in ophthalmology is represented by bevacizumab (Avastin^®^, Roche, Genentech, South San Francisco, CA, USA) for wet age-related macular degeneration (original indication: colorectal cancer). Despite the fact that specific antivirals for SARS-CoV-2 are in development, the drug repurposing approach may suggest additional therapeutics for the ongoing pandemic.

For example, drugs that have demonstrated efficacy in vitro and in animal studies may be included in a panel of broad-spectrum antivirals for emerging viruses. This may be particularly useful in countries under emergency and for healthcare workers, for whom an extension of treatment options is very important during an epidemic.

Several researchers have identified and analyzed the antiviral activity of drugs found in various eye drops and ophthalmic ointments, including coronaviruses.

In this article, we summarize different classes of drugs with antiviral activity that have a repurposing potential (Table 1). Some active ingredients or excipients described here have activity against other types of viruses, thus suggesting potential broad-spectrum applications. Clearly, none of the therapies reported in the present article are suggested for clinical use outside a clinical experimental setting.

In our analysis, seven main categories of drugs contained in topical ophthalmic preparations were considered for their widespread use among patients (excipients, antiseptics, artificial tears, anti-glaucoma medications, antibiotics/antifungals, antiallergic eye drops, and anti-inflammatory ophthalmic preparations).

## 3. Literature Review

A review of the literature for original articles published up to 23 May 2020 was conducted, utilizing Pubmed, Web of Science, Embase, Google Scholar, and Scopus Databases, using the terms “antiviral” and each of the following words relating to a number of topical ophthalmic medications (Bolean operator ‘AND’): “artificial tear”, “antiseptic”, “antibiotic”, “anti-glaucoma”, “antiallergic”, “anti-inflammatory”, “preservatives”, ”buffering agents”, “excipients”, and “antimicrobials”, without any limitation. In addition, manual screening was performed on the reference list of recovered studies for any additional research.

Four independent investigators (P.E.N., L.M., P.G., M.B.) carried out the research. The duplicates have been removed. All titles and abstracts of all citations were analyzed individually. Full texts of the articles deemed potentially eligible were obtained and assessed individually for eligibility.

From 2138 articles retrieved using these searches, we selected 94 papers for our review. In particular, studies that did not focus on the antiviral effect of substances commonly contained in topical ophthalmic preparations for different purposes and documents without new insights into a repurposing potential were excluded.

## 4. Preservatives and Buffering Agents (Excipients)

A level of antimicrobial activity is provided by most eye drops and ophthalmic ointments through the use of preservatives, e.g., in association with artificial tears, anti-glaucoma medications, miotics, anti-inflammatories (nonsteroidal anti-inflammatory drugs, anti-allergics, corticosteroids), or antimicrobial formations (antibacterials, antifungals, antivirals). These chemical additives permit a reduction of ocular infections due to contaminated bottles and a prolonged efficacy of active ingredients or other excipients (i.e., maintaining their functionality and decreasing their biodegradation). For these purposes, oxidants (which deactivate the intracellular enzymes of the microbes and/or alter their nucleic acids, as well as other proteins and lipid components), detergents or surfactants (which non-specifically damage cell membranes), metabolic inhibitors, and chelating agents are commonly used in eye drops [10].

The most widely used preservatives in the market are benzalkonium chloride (BAK; a detergent frequently used in concentration varying from 0.015% to 0.05%), sodium perborate (an oxidative agent that acts by forming hydrogen peroxide), chlorobutanol (detergent), methyl paraben (chelating agent), and stabilized thimerosal (organomercurial). In addition, although disodium-ethylene diamine tetra-acetate (EDTA) and phosphate-buffered saline are not considered as preservatives, they are often included in the formulations of the eye drops as buffering agents [10].

Although an antiviral effect is theoretically possible with all the aforementioned preservatives, those that have so far proven a potential efficacy are as follows: sodium perborate (and related hydrogen peroxide), stabilized oxychloro complex (Purite, an oxidative agent), citric acid (chelating agent/buffering agent), sodium bicarbonate (buffering agent), and boric acid (buffering agent) [11,12,13,14].

Counterintuitively, incongruous data concern quaternary ammonium salt derivates (e.g., BAK) [13,15,16,17,18,19]. In fact, although the latter are generally considered to have virucidal activity against all lipid enveloped viruses [13], some authors claim their inefficiency against SARS-CoV-2 [15,17,19]. In this sense, a question that remains unanswered is as to whether intact RNA alone is an infectious agent, in particular after the COVID-19 virus has lost its envelope due to surfactant-mediated destruction (similar to non-enveloped viruses) [15,17]. Moreover, BAK is a preservative surfactant widely used in eye-drop formulations, but it has many issues regarding its capacity to alter the corneal epithelium, thus potentially increasing permeability of compounds (as active ingredients) and/or infectious agents through this anatomical barrier [11].

On the other hand, thimerosal has been removed from most of the eye drops for its toxic effects on ocular surface epithelia [10].

## 5. Antiseptic-Disinfectant Agents

Disinfectants represent chemical agents mainly used to inactivate or destroy microorganisms on inert surfaces. On the contrary, antiseptics destroy microorganisms on living tissue. Given the lack of specific antiviral treatments for COVID-19, a deeper knowledge of the potential efficacy of available antiseptic disinfectants on viruses is very desirable. Clearly, the time of exposure, the concentration, and the formulation of chemicals are all factors influencing the antiviral action of each agent.

In general, several antiseptic disinfectants are currently recommended to prevent environmental transmission of the COVID-19 virus. Nevertheless, enveloped viruses, such as SARS-CoV-2, are not easily inactivated by different antiseptic disinfectants, such as quaternary ammoniums compounds, hexamidine, phenolic compounds, or chlorhexidine [17].

With regards to the formulations available for ophthalmological use, the most common and potentially interesting are those based on povidone iodine and sodium hypochlorite. Of note, some authors demonstrated that, in suspension tests and for contact times of 5 min, povidone iodine (>0.75% free iodine) can inactivate SARS-CoV-2 infectivity in ~4 log_10_ or more [changes in viral load can be reported as a logarithmic change (in powers of 10) in copies of a specific virus in a defined space], as recommended by the European Standard [19,20,21]. Additionally, sodium hypochlorite (at least 0.21%) has shown efficacy against mouse hepatitis virus (a species of coronavirus) and, consequently, should also be able to inactivate SARS-CoV-2 [19].

The other disinfectants include peroxides and peracids, which promote the production of free radicals that oxidize essential nucleic acids, lipids, and proteins that lead to virucidal activity (e.g., 0.5% hydrogen peroxide with an exposure time of 1 min) [19,20,21,22].

## 6. Artificial Tears

Considering that the prevalence of dry eye disease ranges from 5 to 50% in the general population, it is presumable that this category of eye drops is among the most used by patients. It is interesting to note that a number of substances contained in these formulations (excluding preservatives and buffering agents) are endowed of antiviral action. In particular, we would like to recall the following substances: high molecular weight hyaluronic acid, trehalose, carbopol, and lactoferrin, as well as chamomile oils or extracts of *Echinacea purpurea*, *Rubus fruticosus* (blackberry), *Aloe vera* (i.e., its exudates of aloe emodin), *Ginkgo biloba*, *Centella asiatica*, and *Foeniculum vulgare* (Fennel) [23,24,25,26,27,28,29,30,31,32,33,34,35,36,37,38,39].

Other ingredients presumed to have mild virucidal action are glycerol, l-carnitine, and ozonated oils (O3-Oil) [40,41,42,43,44].

Curiously, excipients commonly classified as antioxidants, i.e., acetylcysteine, vitamin A, vitamin C (ascorbate), have been shown a variable grade of antiviral action [41,45,46,47,48]. Of note, vitamin D has been shown to improve innate or therapeutic antiviral response to various viruses [49,50].

Overall, artificial tears have been shown to possess several mechanisms of antiviral action against a wide range of DNA or RNA viruses [23,24,25,26,27,28,29,30,31,32,33,34,35,36,37,38,39,40,41,42,43,44,45,46,47,48,49,50]. Interestingly, some polymer constituents (e.g., high molecular weight hyaluronic acid) and natural extracts (e.g., *Ginkgo biloba*) have even shown that they can inhibit some viruses belonging to the Coronaviridae family (e.g., porcine parvovirus). In addition, some electrolytes contained in artificial tears with the function of maintaining the ocular surface homeostasis can also have an antiviral effect. For example, zinc (0.25%), which is used as an excipient or in astringent eye drops, has shown that it can inhibit SARS-CoV-2 polymerase activity, blocking viral replication [51].

Considering the large market of this category of eye drops, in particular those based on hyaluronic acid, it is possible to presume that their use may play an important role in protecting the ocular surface from viral infections in a variable number of patients.

On the other hand, topical application of chloroquine may also have a repurposing potential. Chloroquine is an anti-malarial drug with immunomodulatory functions that has demonstrated an antiviral effect through the inhibition of the pH linked steps of viral replication of various viruses (e.g., retroviruses, flaviviruses, and coronaviruses), including SARS-CoV-2. In eye drops, chloroquine has been recently used at a concentration of 0.03% in patients with dry eye [52].

## 7. Anti-Glaucoma Eye Drops

Glaucoma is the leading cause of global irreversible blindness. Present estimates of global glaucoma prevalence are not up-to-date and focus mainly on European ancestry populations. The global prevalence of glaucoma for the population aged 40–80 years is 3.54% and rises progressively by age.

Some drugs used for glaucoma have been shown to affect the clinical course of viral infections. For example, latanoprost has been associated with herpes virus keratitis, potentially increasing the severity and recurrence of the disease [53,54]. Similar behavior has been observed with tafluprost, bimatoprost, and travoprost [55,56,57].

Conversely, timolol maleate acts as an antiviral agent and it is used in the treatment of viral infections, such as herpes simplex infections [58].

Of interest, dorzolamide showed antiviral action against oseltamivir-resistant influenza by an in silico screening, specifically targeting mutant viral neuraminidase [59]. Brinzolamide also showed to be a moderate inhibitor of viral growth of the H3N2 virus and H1N1 influenza viruses and a weak inhibitor of avian H5N2 and H7N1 influenza viruses [60].

Overall, glaucoma drugs may have a synergistic action against viral infections, both for the active ingredients and excipients, but careful monitoring should be suggested for patients treated with prostaglandin analogues.

## 8. Antibiotics and Other Antimicrobials

An antibiotic is a type of antimicrobial agent used to fight and prevent bacterial infections. They can kill and/or inhibit the growth of bacteria. A limited number of antibiotics also have antiviral activity, since viruses do not have cell walls that can be attacked by antibiotics (but a protective protein coat) and cannot reproduce on their own, as bacteria do, having to colonize healthy cells and reprogram them to create new viruses.

Categories of antibiotics with antiviral potential include macrolides (e.g., azithromycin, which demonstrated activity against SARS-CoV-2), tetracyclines (tetracycline, doxycycline, minocycline, which have been proposed in the treatment of COVID-19), fluoroquinolones (e.g., ciprofloxacin, levofloxacin, ofloxacin, which have shown efficacy against polyomavirus BK and influenza virus), aminoglycosides (e.g., on Japanese encephalitis and influenza A virus infection), chloramphenicol (e.g., on human Herpesviridae family), Colistin (polymyxin E, for example on mycobacteriophage D29 infection),and fusidic acid (e.g., on human immunodeficiency virus and John Cunningham virus infection) [61,62,63,64,65,66,67,68,69,70].

Even some antifungals (which are rarely used in ophthalmology) can have a virucidal effect (e.g., amphotericin B, itraconazole, posaconazole) [71,72,73].

## 9. Antiallergic Eye Drops

Allergic eye diseases are another group of pathologies that are very frequent in the general population [74]. It is estimated that around 20% of the world’s population has an allergic disease, of which up to 60% has ocular involvement [75]. For these reasons, antiallergic eye drops represent a large percentage of all eye drops used by patients. The most severe chronic forms, e.g., atopic keratoconjunctivitis, can be debilitating, particularly when associated with a significant tear film dysfunction [76]. Complex responses of the immune system are implicated in the activation and maintenance of these chronic or recurrent inflammatory diseases [77]. Consequently, they may require prolonged treatments with anti-inflammatory agents in the most severe cases.

Surprisingly, several antihistamines, such as chlorcyclizine, chlorpheniramine, and diphenhydramine, have demonstrated antiviral action against hepatitis C virus (HCV), filoviruses (consisting of Ebola virus, Marburg virus, and Cuevavirus), and Influenza A virus infection [78,79,80]. Of note, ketotifen fumarate has proven to attenuate dengue virus infection [81]. Controversial results of antihistamines in antiviral effects appear to be associated with herpes simplex virus (HSV) infection [82].

A group of Flavonoids, plant polyphenols that give flavor and color to vegetables and fruits, has recently gained importance in the pharmaceutical field through its beneficial effects in the prevention or treatment of different ocular diseases, including allergic eye disorders, dry eye disease, diabetic retinopathy, macular degeneration, and cataracts [83,84]. In particular, antiviral effects have been observed against HSV-1, polio-virus type 1, and respiratory syncytial virus (RSV).

Some topical immunomodulators, such as cyclosporine (also used in dry eye therapy), have also shown antiviral action against HCV, Flavivirus and influenza virus [85,86,87].

## 10. Anti-Inflammatory Ophthalmic Preparations

Another interesting category of drugs contained in ophthalmic preparation is that of active substances in reducing inflammation with a broad spectrum of other effects. These drugs are commonly subdivided in two categories: non-steroidal anti-inflammatory drugs (NSAIDs) and steroids. Generally, these eye drops, or ophthalmic ointments, are used to depress or prevent various types of eye inflammations (e.g., during peri-surgical period or for uveitis) or to control ocular pain (e.g., discomfort symptoms after cataract surgery).

Interestingly, some NSAIDs have shown antiviral action. However, a short premise is necessary to understand their function. With regards to the control of recurrences of HSV, latent HSV activation is generally associated with an increase in prostaglandins (PG), which presumably suppress the inhibitory effect of interferon (IFN) on replication HSV. In this sense, indometacin and bromfenac has proven to inhibit HSV-1 replication as they depress PG levels without altering IFN levels [88,89]. On the contrary, steroids may adversely influence, by suppressing the overall inflammatory response, the processes acting to inhibit herpetic viruses. Nevertheless, a short course of topical steroids (e.g., dexamethasone) may be useful for patients with acute, presumed, and aspecific viral conjunctivitis [90,91,92].

## 11. Discussion and Conclusions

Although systemic and topical (eye drops/ophthalmic ointments) antiviral medications have been utilized in a small number of cases by different authors, no specific antivirals are currently available for SARS-CoV-2 associated conjunctivitis. Overall, this pathology of the ocular surface still leaves many questions unanswered [3], being associated with some controversial and unclear data.

First, a patient with this conjunctivitis as an earlier symptom may have a negative conjunctival sac SARS-CoV-2 test, but also the opposite may be observed. According to a recent meta-analysis on 1167 patients, the overall rate of conjunctivitis appears to be 1.1% (13/1149), of which 3% (6/195) and 0.7% (7/954) was found in severe and non-severe forms of COVID-19, respectively [93]. Nevertheless, an increasing number of conjunctivitis case reports are continuously appearing in literature.

Second, SARS-CoV-2 conjunctivitis has been described as a mild follicular conjunctivitis otherwise indistinguishable from other viral causes [94]. In addition, other features of ocular surface involvement include unilateral or bilateral bulbar conjunctiva hyperemia alone or in association with chemosis, follicular reaction of the palpebral conjunctiva, watery discharge, epiphora, and mild eyelid edema.

Another unresolved question is whether conjunctivitis is directly related to virus infection or represents an allergic immune response to the virus [95].

Generally, viral conjunctivitis (of unknown origin) does not require treatment, although antibiotics, steroids, and artificial tear drops are often prescribed to relieve inflammation signs and symptoms. Most medicines currently available for the treatment of viral conjunctivitis are directed against herpes and adenovirus infections, and infectious diseases of the eye caused by RNA viruses (such as influenza, RSV, or coronavirus) lack targeted antiviral medications. Skevaki et al. recommend the use of oseltamivir, ganciclovir, and other drugs for treatment and prevention during the onset of conjunctivitis symptoms or a history of eye contact [96].

According to Zhou [97], the rarity of viral conjunctivitis in SARS-CoV-2 infection may exist in three interpretations. Firstly, the expression of the ACE2 protein on conjunctival epithelial cell membranes is much less than that in human lung and kidney tissues [8,9]. Secondly, the binding capability of the ACE2 protein on conjunctival epithelial cells to SARS-CoV spike protein is much lower than that in lung tissues [98]. Thirdly, the protective effect of the antimicrobial agents in tears, including lactoferrin and secretory IgA, and constant tear rinsing on the ocular surface, which could eliminate the viruses, dropped onto the ocular surface and into the nasal cavity through the nasolacrimal duct [3].

However, the present study shows that a large proportion of ophthalmic preparations, utilized for different ocular diseases, contain substances with an intrinsic, broad spectrum, antiviral activity, which has already been used clinically in the past for other (non-COVID-19) viral infections. In particular, here we want to emphasize the potential usefulness of artificial tears and iodine/sodium hypochlorite eye drops to promote the reduction of viral load on the ocular surface by removing the virus or by means of a direct virucidal action.

A very interesting aspect to note is that a large portion of these eye drops, or ophthalmic ointments are registered as over-the-counter (OTC). As such, they can be easily and widely purchased by patients without the need for a prescription or an ophthalmological consultation. For example, artificial tears are not only sold in pharmacies, but they are often found in supermarkets or optical stores.

Clearly, the use of any eye drops (e.g., saline solution) may also facilitate the reduction of viral load through the washing of infectious agents [99]. Accordingly, the excretion of elements present in human tears towards the lid skin occurs not only in overflow conditions due to a large volume, but also because of a mechanism related to the characteristics of high fluid dynamics (turbulence of the meniscus) [100,101]. Of note, the volume of a drop obtained from a bottle of ophthalmic solution generally ranges from 25 to 70 µL, while the ocular surface may contain much less liquid [102]. For all these reasons, each administration of eye drops implies a wash-out of any substances above the epithelia of the ocular surface.

As a comparison, a different concept is that concerning ophthalmic ointments. In fact, the latter do not favor the washing of infectious agents but can release drugs for a long time. This aspect can be advantageous if the substance has an antiviral effect (a presumably common situation), while in the case of steroid ointments, for example, it could be very disadvantageous with detrimental effects on the ocular surface epithelia (e.g., following herpes virus infection).

It is possible that a fraction of patients affected by COVID-19 regularly use any type of these topical medications, suggesting an alternative explanation for the limited detection of viruses in tears and on the ocular surface observed in some series of COVID-19 patients. Specific studies might enlighten on this hypothesis.

At present, the drug repurposing approach is widely used to identify potential therapies for different diseases. In recent years, it has gained a lot of popularity in the scientific community for the ability to reuse drugs already available for various diseases beyond their original indication. This approach is based on the principle that different diseases share overlapping molecular pathways and various drugs have multiple protein targets [103]. The advantages of reusing approved drugs in this way include reducing costs, time, and risks associated with the experimental phases of *de novo* drugs [104]. Clearly, the drug development approach (e.g., regarding virus-specific vaccines or small molecules) is inadequate to immediately address the problems associated with a pandemic such as COVID-19. The concept of redefining the potentiality of existing drugs is one that most quickly meets the needs of a population under a health emergency.

It should be noted that, if the use of such drugs re-proposed individually may be clinically ineffective, their carefully evaluated combinations could benefit patients, which happened with human immunodeficiency virus (HIV) during the 1990s [105]. In this sense, various eye drops simultaneously contain several substances with antiviral action, e.g., active ingredients, buffering agents, preservatives, or other excipients. In the future, one aspect that will determine the effectiveness of this drug repurposing strategy will be the comparison, positive or negative, with the treatments developed specifically for COVID-19 (in particular, vaccines or drugs). In fact, a feature of these repurposed drugs is their non-specificity for an infectious agent (e.g., SARS-CoV-2), which can become a significant problem in the case of sudden drug resistance or more virulent strains. However, the discovery of vaccines and antibodies generally takes up to a decade and may be slowed by the potential for attenuated antigenicity of epitopes due to the genetic drift of the virus.

Overall, the results of this work strongly suggest that ophthalmic preparations represent a vast reservoir of potential candidates for drug repurposing for use as antiviral therapeutics. Here, we have analyzed only the main categories of substances contained in ophthalmic preparations with re-profiling potential. However, there are also other groups of drugs, such as antihypertensives and anticoagulants, which might unexpectedly give interesting results by topical application (e.g., ACE inhibitors, or heparin sodium) [106,107,108]. In this sense, tea tree oil (TTO) is another therapeutic option for viral infections, which is widely used in gel formulations to be applied on the eyelid skin (not inside the eye). In several studies, TTO has demonstrated anti-viral action against HSV, Influenza virus A/PR/8, and Tobacco Mosaic virus (a Tobamovirus that infects a wide range of plants) [109,110,111].

In addition, particular attention is recommended in the follow-up of glaucomatous patients treated with prostaglandin analogues or uveitic patients under steroids, for which the use of lubricants is particularly indicated for different reasons, e.g., limiting in situ virus replication and epithelial damage.

Clearly, all information reported in this article is not intended to guide clinical decisions, and any potential therapy reported here should only be considered as a proposal to be evaluated in the context of clinical trials.

## Figures and Tables

**Table 1:** A panel of broad-spectrum antivirals in topical eye medications from the drug repurposing approach.

	Original Indication	Repurposing Potential as Antiviral	*Note*
Benzalkonium Chloride (BAK) (0.015–0.05%)	Preservative (Detergent)	e.g., DNA (HSV-2, CMV, Adenovirus, BK Virus) and RNA (RSV, Enterovirus, Norovirus, Porcine Epidemic Diarrhea) Viruses	Controversial Results for SARS-Cov-2 (See Text)
Chlorobutanol	Preservative (Detergent)	e.g., HBV	-
Sodium Perborate (And Related Hydrogen Peroxide)	Preservative (Oxidative)	Broad Antiviral Effect	-
Stabilized Oxychloro Complex (SOC) (Purite^®^, Bio-Cide International Inc., Norman, OK, USA)	Preservative (Oxidative)	Broad Antiviral Effect	Balancing Antimicrobial Efficacy and Toxicity of Currently Available Topical Ophthalmic Preservatives
Methyl Paraben	Preservative (Chelating Agent)	Antiretroviral Effect	-
Citric Acid	Preservative (Chelating Agent)/*Buffering Agent*	e.g., Coxsackievirus, Herpesviruses, Porcine Epidemic Diarrhea Virus (Coronavirus)	-
Thimerosal	Preservative (Organomercurial)	e.g., Pseudorabies Virus	Removed from Ophthalmic Preparations for Toxic Effects
Disodium-Ethylene Diamine Tetra-Acetate (EDTA)	*Buffering Agents*	e.g., Porcine Epidemic Diarrhea Virus (Coronavirus)	-
Phosphate-Buffered Saline	*Buffering Agents*	e.g., Porcine Epidemic Diarrhea Virus (Coronavirus)	-
Sodium Bicarbonate	*Buffering Agents*	e.g., Calicivirus	-
Boric Acid	*Buffering Agents*	e.g., White Spot Syndrome Virus (WSSV)	-
Povidone Iodine (<0.76% Free Iodine)	Antiseptic Agent	SARS-Cov-2 and Others	In Suspension Tests and Contact Times of 5 Min
Sodium Hypochlorite (At Least 0.21%)	Antiseptic Agent	e.g., MHV	-
Chlorhexidine	Antiseptic Agent	e.g., Coronaviruses	Modest Antiviral Action
Hexamidine	Antiseptic Agent	e.g., Coronaviruses	Weak Antiviral Action
Polyhexamethylene Biguanide (PHMB)	Antiseptic Agent	e.g., HIV, Herpesviruses, HPV	-
Phenolic Compounds	Antiseptic Agent	Broad Antiviral Effect	Weak Antiviral Action
High Molecular Weight Hyaluronic Acid	Artificial Tear	Coxsackievirus, Influenza Virus, HSV-1, Porcine Parvovirus (Coronavirus)	Mild Inhibition Of HSV-1 and Porcine Parvovirus
Trehalose	Artificial Tear	e.g., EMC Virus	-
Carbopol	Artificial Tear	e.g., HSV-1/HSV-2, HZV	-
Lactoferrin	Artificial Tear	e.g., HIV, CMV	-
Chamomile Oils	Artificial Tear (*Vegetal Extract*)	e.g., Herpesviruses	-
*Echinacea Purpurea*	Artificial Tears (*Vegetal Extract*)	e.g., HSV-1	-
*Rubus Fruticosus (Blackberry)*	Artificial Tears (*Vegetal Extract*)	e.g., HSV-1	-
*Ginkgo Biloba*	Artificial Tears (*Vegetal Extract*)	e.g., Influenza A H3N2, HBV, Porcine Parvovirus (Coronavirus)	-
*Centella Asiatica*	Artificial Tears (*Vegetal Extract*)	e.g., HSV-1, Vesicular Stomatitis Viruses (VSV)	-
*Foeniculum Vulgare*	Artificial Tears (*Vegetal Extract*)	e.g., Bluetongue Virus	-
*Aloe Vera*	Artificial Tears (*Vegetal Extract*)	e.g., SARS-CoV, CMV, Enterovirus 71, Japanese Encephalitis Virus, Herpesviruses, Influenza A Virus, Haemagglutinating Viruses	*Aloe Emodin* is an Anthraquinone and a Variety of Emodin Present in Aloe Latex, An Exudate From the Aloe Plant. In Some Cases, *Aloe Emodin* is Obtained and Studied After Extraction from the *Isatis Indigotica*
Glycerol	Artificial Tears (Excipient)	e.g., HIV-1	-
L-Carnitine	Artificial Tears (Excipient)	e.g., HIV-1	-
Ozonated Oils	Artificial Tears (Excipients)	e.g., Plant Viruses, Coronavirus	Presumed Antiviral Action on SARS-Cov-2 Based on Oxidation of Specific Viral Receptors in Cellular Plants
Zinc	Artificial Tears (Excipient)/Astringent Eye Drops	SARS-Cov-2	Blocking of Viral Replication by Inhibiting SARS-Cov-2 Polymerase Activity
Acetylcysteine	Artificial Tears (Antioxidant)	e.g., HIV-1	-
Vitamin A	Artificial Tears (Antioxidant)	e.g., Norovirus	-
Vitamin C	Artificial Tears (Antioxidant)	e.g., Herpesviruses	-
Vitamin D	Artificial Tears (Immunomodulator)	e.g., HCV	By Improving Innate or Therapeutic Antiviral Response to Various Viruses
Chloroquine	Artificial Tears (Immunomodulator)	SARS-Cov-2, Retroviruses, Flaviviruses, And Coronaviruses	Inhibition of the Ph Linked Steps of Viral Replication
Timolol Maleate	Anti-Glaucoma Eye Drops	e.g., HSV-1	-
Dorzolamide	Anti-Glaucoma Eye Drops	e.g., Influenza Viruses	-
Brinzolamide	Anti-Glaucoma Eye Drops	e.g., H3N2, H1N1, Avian H5N2, H7N1 Influenza Viruses	-
Azithromycin	Antibiotic	SARS-Cov-2 Infection	-
Tetracyclines	Antibiotic	SARS-Cov-2 Infection	-
Fluoroquinolones	Antibiotic	e.g., Influenza Virus, Polyomavirus BK	-
Aminoglycosides	Antibiotic	e.g., Influenza A Virus, Japanese Encephalitis Virus	-
Chloramphenicol	Antibiotic	e.g., Herpetic Stomatitis, Herpes Labialis	-
Colistin	Antibiotic	e.g., Mycobacteriophage D29	-
Fusidic Acid	Antibiotic	e.g., HIV, JC Virus	-
Itraconazole	Antifungal	e.g., Parechovirus A3 (Picornaviridae), Influenza Virus	-
Posaconazole	Antifungal	e.g., ParechovirusA3 (Picornaviridae)	-
Amphotericin B	Antifungal	e.g., Vesicular Stomatitis Virus, HSV-1, HSV-2, Sindbis Virus, Vaccinia Virus	-
Ketotifen Fumarate	Anti-Allergic	e.g., Dengue Virus	Controversial Results for Herpesviruses (See Text)
Chlorcyclizine	Anti-Allergic	e.g., HCV, Filoviridae (Ebola Virus, Marburg Virus and Cuevavirus)	-
Chlorpheniramine	Anti-Allergic	e.g., Influenza A Virus	-
Diphenhydramine	Anti-Allergic	e.g., Filoviridae (Ebola Virus, Marburg Virus and Cuevavirus)	-
Flavonoids	Anti-Allergic/Artificial Tear	e.g., HSV-1, Polio-Virus Type 1, Respiratory Syncytial Virus (RSV)	Broad Effect on Eye Disease (See Text)
Cyclosporine	Anti-Allergic	e.g., HCV, Flavivirus, Influenza Virus	Broad Effect on Inflammatory Eye Disease (See Text)
Indometacin	Non-Steroidal Anti-Inflammatory Drugs (NSAIDs)	e.g., HSV-1	-
Bromfenac	Non-Steroidal Anti-Inflammatory Drugs (NSAIDs)	e.g., HSV-1	-

Different types of active ingredients and excipients commonly contained in ophthalmic preparations that show antiviral side effects against various viruses (in addition to their primary use). These substances can have a broad-spectrum action, possibly including inhibition of replication/diffusion of severe acute respiratory syndrome coronavirus 2 (SARS-CoV-2) on the ocular surface. Abbreviations were as follows: Herpes simplex virus (HSV); Herpes simplex virus type 1 and/or 2 (HSV-1 and/or HSV-2); Herpes zoster virus (HZV); Cytomegalovirus (CMV); Hepatitis B virus (HBV); Hepatitis C virus (HCV); Human immunodeficiency virus (HIV); Human papillomavirus (HPV); BK virus: this infectious agent is a member of the polyomavirus family (it was first isolated in 1971 from the urine of a kidney transplant patient; initials B.K.); John Cunningham virus (JC virus or Human polyomavirus 2); Mycobacteriophage D29: this infectious agent is a Cluster A mycobacteriophage, belonging to the *Siphoviridae* family of viruses (notable for its ability to infect *M. tuberculosis*); Mouse hepatitis virus (MHV); Encephalomyocarditis virus (EMC).H_(X)_N_(Y)_: Influenza A viruses. Based on two proteins on the virus surface, i.e., hemagglutinin (H) and neuraminidase (N), these infectious agents are classified into different subtypes: H1N1 (the common cause of human influenza and generally associated with the Spanish flu), H3N2 (commonly associated with swine flu or seasonal H3N2 flu), H5N2 or H7N1 (generally associated with avian influenza virus or bird flu virus). e.g., -exempli gratia.

## References

[1] Napoli P.E., Nioi M. (2020). Global Spread of Coronavirus Disease 2019 and Malaria: An Epidemiological Paradox in the Early Stage of a Pandemic. J. Clin. Med..

[2] Napoli P.E., Nioi M., D’Aloja E., Fossarello M. (2020). Safety Recommendations and Medical Liability in Ocular Surgery during the COVID-19 Pandemic: An Unsolved Dilemma. J. Clin. Med..

[3] Napoli P.E., Nioi M., D’Aloja E., Fossarello M. (2020). The Ocular Surface and the Coronavirus Disease 2019: Does a Dual ‘Ocular Route’ Exist?. J. Clin. Med..

[4] Xia J., Tong J., Liu M., Shen Y., Guo D. (2020). Evaluation of coronavirus in tears and conjunctival secretions of patients with SARS-CoV-2 infection. J. Med. Virol..

[5] Lu C.-W., Liu X.-F., Jia Z.-F. (2020). 2019-nCoV transmission through the ocular surface must not be ignored. Lancet.

[6] Li X.J., Wang M., Dai J., Wang W., Yang Y., Jin W. (2020). Novel coronavirus disease with conjunctivitis and conjunctivitis as first symptom: Two cases report. Chin. J. Exp. Ophthalmol..

[7] Li X.J., Wang M., Chen C.Z., Yang A., Jin W. (2020). Ophthalmologists’ strategy for the prevention and control of coronavirus pneumonia with conjunctivitis or with conjunctivitis as the first symptom. Chin. J. Exp. Ophthalmol..

[8] Guan W.-J., Ni Z.-Y., Hu Y., Liang W.-H., Ou C.-Q., He J.-X., Liu L., Shan H., Lei C.-L., Hui D.S. (2020). Clinical Characteristics of Coronavirus Disease 2019 in China. N. Engl. J. Med..

[9] Wu P., Duan F., Luo C., Liu Q., Qu X., Liang L., Wu K. (2020). Characteristics of Ocular Findings of Patients With Coronavirus Disease 2019 (COVID-19) in Hubei Province, China. JAMA Ophthalmol..

[10] Epstein S.P., Ahdoot M., Marcus E., Asbell P.A. (2009). Comparative Toxicity of Preservatives on Immortalized Corneal and Conjunctival Epithelial Cells. J. Ocul. Pharmacol. Ther..

[11] Tu E.Y. (2014). Balancing antimicrobial efficacy and toxicity of currently available topical ophthalmic preservatives. Saudi J. Ophthalmol..

[12] Malik Y.S., Goyal S.M. (2006). Virucidal efficacy of sodium bicarbonate on a food contact surface against feline calicivirus, a norovirus surrogate. Int. J. Food Microbiol..

[13] List N: Disinfectants for Use against SARS-CoV-2. https://www.epa.gov/pesticide-registration/list-n-disinfectants-use-against-sars-cov-2.

[14] Hernandez-Patlan D., Solis-Cruz B., Méndez-Albores A., Latorre J.D., Hernandez-Velasco X., Tellez-Isaias G., López-Arellano R. (2018). Comparison of PrestoBlue^®^ and plating method to evaluate antimicrobial activity of ascorbic acid, boric acid and curcumin in an in vitro gastrointestinal model. J. Appl. Microbiol..

[15] Schrank C.L., Minbiole K.P.C., Wuest W.M. (2020). Are Quaternary Ammonium Compounds, the Workhorse Disinfectants, Effective against Severe Acute Respiratory Syndrome-Coronavirus-2?. ACS Infect. Dis..

[16] Wood A., Payne D. (1998). The action of three antiseptics/disinfectants against enveloped and non-enveloped viruses. J. Hosp. Infect..

[17] Sattar S.A., Springthorpe V.S., Karim Y., Loro P. (1989). Chemical disinfection of non-porous inanimate surfaces experimentally contaminated with four human pathogenic viruses. Epidemiol. Infect..

[18] Rabenau H., Kampf G., Cinatl J., Doerr H. (2005). Efficacy of various disinfectants against SARS coronavirus. J. Hosp. Infect..

[19] Kampf G., Todt D., Pfaender S., Steinmann E. (2020). Persistence of coronaviruses on inanimate surfaces and their inactivation with biocidal agents. J. Hosp. Infect..

[20] Sizun J., Yu M., Talbot P. (2000). Survival of human coronaviruses 229E and OC43 in suspension and after drying onsurfaces: A possible source ofhospital-acquired infections. J. Hosp. Infect..

[21] Kariwa H., Fujii N., Takashima I. (2006). Inactivation of SARS Coronavirus by Means of Povidone-Iodine, Physical Conditions and Chemical Reagents. Dermatology.

[22] Chen C.-J., Ding S.-J. (2016). Effectiveness of Hypochlorous Acid to Reduce the Biofilms on Titanium Alloy Surfaces in Vitro. Int. J. Mol. Sci..

[23] Cermelli C., Cuoghi A., Scuri M., Bettua C., Neglia R.G., Ardizzoni A., Blasi E., Iannitti T., Palmieri B. (2011). In vitro evaluation of antiviral and virucidal activity of a high molecular weight hyaluronic acid. Virol. J..

[24] Guillemard E., Geniteau-Legendre M., Mabboux B., Poilane I., Kergot R., Lemaire G., Petit J., Labarre C., Quero A. (1993). Antiviral action of trehalose dimycolate against EMC virus: Role of macrophages and interferon α/β. Antivir. Res..

[25] Guillemard E., Geniteau-Legendre M., Kergot R., Lemaire G., Petit J., Labarre C., Quero A. (1995). Role of trehalose dimycolate-induced interferon-α/β in the restriction of encephalomyocarditis virus growth in vivo and in peritoneal macrophage cultures. Antivir. Res..

[26] De Clercq E., Luczak M. (1976). Antiviral activity of carbopol, a cross-linked polycarboxylate. Arch. Virol..

[27] Harmsen M.C., Swart P.J., Bethune M.-P.D., Pauwels R., Clercq E.D., The T.B., Meijer D.K.F. (1995). Antiviral Effects of Plasma and Milk Proteins: Lactoferrin Shows Potent Activity against Both Human Immunodeficiency Virus and Human Cytomegalovirus Replication In Vitro. J. Infect. Dis..

[28] Van Der Strate B.W., Beljaars L., Molema G., Harmsen M.C., Meijer D. (2001). Antiviral activities of lactoferrin. Antivir. Res..

[29] Koch C., Reichling J., Schneele J., Schnitzler P. (2008). Inhibitory effect of essential oils against herpes simplex virus type 2. Phytomedicine.

[30] Koch C., Reichling J., Kehm R., Sharaf M.M., Zentgraf H., Schneele J., Schnitzler P. (2008). Efficacy of anise oil, dwarf-pine oil and chamomile oil against thymidine-kinase-positive and thymidine-kinase-negative herpes viruses. J. Pharm. Pharmacol..

[31] Binns S.E., Merali S., Hudson J., Arnason J.T. (2002). Antiviral Activity of Characterized Extracts from Echinacea spp. (Heliantheae: Asteraceae) against Herpes simplex Virus (HSV-I). Planta Med..

[32] Danaher R.J., Wang C., Dai J., Mumper R.J., Miller C.S. (2011). Antiviral effects of blackberry extract against herpes simplex virus type 1. Oral Surg. Oral Med. Oral Pathol. Oral Radiol. Endodontol..

[33] Lin L.-T., Hsu W.-C., Lin C.-C. (2014). Antiviral Natural Products and Herbal Medicines. J. Tradit. Complement. Med..

[34] Wang C.-Z., Li W.-J., Tao R., Ye J.-Z., Zhang H.-Y. (2015). Antiviral Activity of a Nanoemulsion of Polyprenols from Ginkgo Leaves against Influenza A H3N2 and Hepatitis B Virus in Vitro. Molecules.

[35] Lee J.-H., Park J.-S., Lee S.-W., Hwang S.-Y., Young B.-E., Choi H.-J. (2015). Porcine epidemic diarrhea virus infection: Inhibition by polysaccharide from Ginkgo biloba exocarp and mode of its action. Virus Res..

[36] Hamidi J.A., Ismaili N.H., Ahmadi F.B., Lajisi N.H. (1996). Antiviral and cytotoxic activities of some plants used in Malaysian indigenous medicine. Pertanika J. Trop. Agric. Sci..

[37] Tharanath V., Peddanna K., Kotaiah Y., Venkataramana D. (2013). Flavonoids isolated from Foeniculum vulgare (Fennel) have virostatic efficiency against bluetongue virus. Int. J. Pharm. Sci. Rev. Res..

[38] Reichling J., Schnitzler P., Suschke U., Saller R. (2009). Essential Oils of Aromatic Plants with Antibacterial, Antifungal, Antiviral, and Cytotoxic Properties—An Overview. Complement. Med. Res..

[39] De Clercq E. (2014). Potential antivirals and antiviral strategies against SARS coronavirus infections. Expert Rev. Anti-Infect. Ther..

[40] Welch J.L., Xiang J., Okeoma C.M., Schlievert P.M., Stapleton J.T. (2020). Glycerol Monolaurate, an Analogue to a Factor Secreted by Lactobacillus, Is Virucidal against Enveloped Viruses, Including HIV-1. mBio.

[41] Patrick L. (2000). N-acetylcysteine, Alpha-Lipoic Acid, L-Glutamine, and L-Carnitine. Altern. Med. Rev..

[42] Travagli V., Zanardi I., Bocci V. (2009). Topical applications of ozone and ozonated oils as anti-infective agents: An insight into the patent claims. Recent Pat. Anti-Infect. Drug Discov..

[43] Ugazio E., Tullio V., Binello A., Tagliapietra S., Dosio F. (2020). Ozonated Oils as Antimicrobial Systems in Topical Applications. Their Characterization, Current Applications, and Advances in Improved Delivery Techniques. Molecules.

[44] Rowen R.J., Robins H. (2020). A Plausible “Penny” Costing Effective Treatment for Corona Virus Ozone Therapy. J. Infect. Dis. Epidemiol..

[45] Lee H., Ko G. (2016). Antiviral effect of vitamin A on norovirus infection via modulation of the gut microbiome. Sci. Rep..

[46] Koyama A.H., Furuya A., Uozaki M., Yamasaki H., Arakawa T., Arita M. (1998). Antiviral effects of ascorbic and dehydroascorbic acids in vitro. Int. J. Mol. Med..

[47] Brinkevich S.D., Boreko E., Savinova O.V., Pavlova N.I., Shadyro O.I. (2012). Radical-regulating and antiviral properties of ascorbic acid and its derivatives. Bioorg. Med. Chem. Lett..

[48] Hovi T., Hirvimies A., Stenvik M., Vuola E., Pippuri R. (1995). Topical treatment of recurrent mucocutaneous herpes with ascorbic acid-containing solution. Antivir. Res..

[49] Bitetto D., Fabris C., Fornasiere E., Pipan C., Fumolo E., Cussigh A., Bignulin S., Cmet S., Fontanini E., Falleti E. (2010). Vitamin D supplementation improves response to antiviral treatment for recurrent hepatitis C. Transpl. Int..

[50] Gal-Tanamy M., Bachmetov L., Ravid A., Koren R., Erman A., Tur-Kaspa R., Zemel R. (2011). Vitamin D: An innate antiviral agent suppressing hepatitis C virus in human hepatocytes. Hepatology.

[51] Hubbard G.B., Herron B.E., Andrews J.S., Elliott J.H. (1969). Influence of topical and oral zinc upon corneal wound healing. Br. J. Ophthalmol..

[52] Titiyal J.S., Kaur M., Falera R., Bharghava A., Sah R., Sen S. (2019). Efficacy and Safety of Topical Chloroquine in Mild to Moderate Dry Eye Disease. Curr. Eye Res..

[53] Wand M., Gilbert C.M., Liesegang T.J. (1999). Latanoprost and herpes simplex keratitis. Am. J. Ophthalmol..

[54] Kaufman H.E., Varnell E.D., Thompson H.W. (1999). Latanoprost increases the severity and recurrence of herpetic keratitis in the rabbit. Am. J. Ophthalmol..

[55] Park H.G., Choi S. (2013). A Case of Herpetic Simplex Keratitis after Application of 0.015% Tafluprost Eye Drops. J. Korean Ophthalmol. Soc..

[56] Kroll D.M., Schuman J.S. (2002). Reactivation of herpes simplex virus keratitis after initiating bimatoprost treatment for glaucoma. Am. J. Ophthalmol..

[57] Yang H.S., Park H.G., Choi S. (2011). Reactivation of Herpetic Keratitis in a Patient after Using Two Different Prostaglandin Analogues. J. Korean Ophthalmol. Soc..

[58] Shanmugam S., Vetrichelvan T. (2017). Formulation Development and Evaluation of OpthalmicOcusert Containing Timolol Maleate. Res. Rev. A J. Drug Formul. Dev. Prod..

[59] Bao J., Marathe B., Govorkova E.A., Zheng J.J. (2016). Drug Repurposing Identifies Inhibitors of Oseltamivir-Resistant Influenza Viruses. Angew. Chem. Int. Ed. Engl..

[60] Josset L., Textoris J., Loriod B., Ferraris O., Moules V., Lina B., N’Guyen C., Diaz J.-J., Rosa-Calatrava M. (2010). Gene Expression Signature-Based Screening Identifies New Broadly Effective Influenza A Antivirals. PLoS ONE.

[61] Juurlink D.N. (2020). Safety considerations with chloroquine, hydroxychloroquine and azithromycin in the management of SARS-CoV-2 infection. Can. Med. Assoc. J..

[62] Sodhi M., Etminan M. (2020). Therapeutic Potential for Tetracyclines in the Treatment of COVID-19. Pharmacother. J. Hum. Pharmacol. Drug Ther..

[63] Enoki Y., Ishima Y., Tanaka R., Sato K., Kimachi K., Shirai T., Watanabe H., Chuang V.T.G., Fujiwara Y., Takeya M. (2015). Pleiotropic Effects of Levofloxacin, Fluoroquinolone Antibiotics, against Influenza Virus-Induced Lung Injury. PLoS ONE.

[64] Knoll G.A., Humar A., Fergusson D., Johnston O., House A.A., Kim S.J., Ramsay T., Chassé M., Pang X., Zaltzman J. (2014). Levofloxacin for BK Virus Prophylaxis Following Kidney Transplantation. JAMA.

[65] Kim H., Lee M.-K., Ko J., Park C.-J., Kim M., Jeong Y., Hong S., Varani G., Choi B.-S. (2012). Aminoglycoside antibiotics bind to the influenza a virus RNA promoter. Mol. BioSyst..

[66] Topno R., Khan S.A., Chowdhury P., Mahanta J. (2016). Pharmacodynamics of aminoglycosides and tetracycline derivatives against Japanese encephalitis virus. Asian Pac. J. Trop. Med..

[67] Zegarelli E.V., Budowsky J., Silvers H.F., Kutscher A.H. (1953). Chloramphenicol in Treatment of Primary Herpetic Stomatitis And Herpes Labialis. Arch. Dermatol..

[68] David H.L., Rastogi N., Clavel-Sérès S., Clément F. (1986). Action of Colistin (Polymyxin E) on the Lytic Cycle of the Mycobacteriophage D29 in Mycobacterium tuberculosis. Zent. Bakteriol. Mikrobiol. Hyg. Ser. A Med. Microbiol. Infect. Dis. Virol. Parasitol..

[69] Chan J.F.-W., Ma M.K.-M., Chan G.S.-W., Chan G.C.-W., Choi G.K.-Y., Chan K.-H., Cheng V.C.-C., Choy B.-Y., Yuen K.-Y., Chan K.-W. (2015). Rapid reduction of viruria and stabilization of allograft function by fusidic acid in a renal transplant recipient with JC virus-associated nephropathy. Infection.

[70] Famularo G., De Simone C., Tzantzoglou S., Trinchieri V., Moretti S., Tonietti G. (1993). In Vivo and in Vitro Efficacy of Fusidic Acid in HIV Infection. Ann. N. Y. Acad. Sci..

[71] Rhoden E., Nix W.A., Weldon W.C., Selvarangan R. (2018). Antifungal azoles itraconazole and posaconazole exhibit potent in vitro antiviral activity against clinical isolates of parechovirus A3 (Picornaviridae). Antivir. Res..

[72] Jordan G.W., Seet E.C. (1978). Antiviral Effects of Amphotericin B Methyl Ester. Antimicrob. Agents Chemother..

[73] Schloer S., Goretzko J., Kühnl A., Brunotte L., Ludwig S., Rescher U. (2019). The clinically licensed antifungal drug itraconazole inhibits influenza virus in vitro and in vivo. Emerg. Microbes Infect..

[74] Takamura E., Uchio E., Ebihara N., Ohno S., Ohashi Y., Okamoto S., Kumagai N., Satake Y., Shoji J., Nakagawa Y. (2017). Japanese guidelines for allergic conjunctival diseases 2017. Allergol. Int..

[75] Leonardi A., Bogacka E., Fauquert J.-L., Kowalski M.L., Groblewska A., Jedrzejczak-Czechowicz M., Doan S., Marmouz F., Demoly P., Delgado L. (2012). Ocular allergy: Recognizing and diagnosing hypersensitivity disorders of the ocular surface. Allergy.

[76] Bron A.J., De Paiva C.S., Chauhan S.K., Bonini S., Gabison E.E., Jain S., Knop E., Markoulli M., Ogawa Y., Perez V. (2017). TFOS DEWS II pathophysiology report. Ocul. Surf..

[77] Zhai J., Gu J., Yuan J., Chen J.-Q. (2011). Tacrolimus in the Treatment of Ocular Diseases. BioDrugs.

[78] He S., Lin B., Chu V., Hu Z., Hu X., Xiao J., Wang A.Q., Schweitzer C.J., Li Q., Imamura M. (2015). Repurposing of the antihistamine chlorcyclizine and related compounds for treatment of hepatitis C virus infection. Sci. Transl. Med..

[79] Xu W., Xia S., Pu J., Wang Q., Li P., Lu L., Jiang S. (2018). The Antihistamine Drugs Carbinoxamine Maleate and Chlorpheniramine Maleate Exhibit Potent Antiviral Activity against a Broad Spectrum of Influenza Viruses. Front. Microbiol..

[80] Schafer A., Cheng H., Xiong R., Soloveva V., Retterer C., Mo F., Bavari S., Thatcher G., Rong L. (2018). Repurposing potential of 1st generation H 1 -specific antihistamines as anti-filovirus therapeutics. Antivir. Res..

[81] Tan J.W., Zahidi N.F.W., Kow A.S.F., Soo K.M., Shaari K., Israf D.A., Chee H.Y., Tham C.L. (2019). Mast cell stabilizing effect of a geranyl acetophenone in dengue virus infection using in vitro model of DENV3-induced RBL-2H3 cells. Biosci. Rep..

[82] Zawar V.P., Godse K., Sankalecha S. (2010). Chronic urticaria associated with recurrent genital herpes simplex infection and success of antiviral therapy—A report of two cases. Int. J. Infect. Dis..

[83] Chan S.-C., Chang Y.-S., Wang J.-P., Chen S.-C., Kuo S.-C. (1998). Three New Flavonoids and Antiallergic, Anti-Inflammatory Constituents from the Heartwood of Dalbergia odorifera. Planta Med..

[84] Kaul T.N., Middleton E., Ogra P.L. (1985). Antiviral effect of flavonoids on human viruses. J. Med. Virol..

[85] Rabie R., Mumtaz K., Renner E.L. (2012). Efficacy of antiviral therapy for hepatitis C after liver transplantation with cyclosporine and tacrolimus: A systematic review and meta-analysis. Liver Transplant..

[86] Qing M., Yang F., Zhang B., Zou G., Robida J.M., Yuan Z., Tang H., Shi P.-Y. (2009). Cyclosporine Inhibits Flavivirus Replication through Blocking the Interaction between Host Cyclophilins and Viral NS5 Protein. Antimicrob. Agents Chemother..

[87] Ma C., Li F., Musharrafieh R.G., Wang J. (2016). Discovery of cyclosporine A and its analogs as broad-spectrum anti-influenza drugs with a high in vitro genetic barrier of drug resistance. Antivir. Res..

[88] Khyatti M., Menezes J. (1990). The effect of indometacin, prostaglandin E2 and interferon on the multiplication of herpes simplex virus type 1 in human lymphoid cells. Antivir. Res..

[89] Higaki S., Watanabe K., Itahashi M., Shimomura Y. (2009). Cyclooxygenase (COX)-Inhibiting Drug Reduces HSV-1 Reactivation in the Mouse Eye Model. Curr. Eye Res..

[90] Kaye S., Choudhary A. (2006). Herpes simplex keratitis. Prog. Retin. Eye Res..

[91] Wilkins M.R., Khan S., Bunce C., Khawaja A.P., Siriwardena D., Larkin D.F.P. (2011). A randomised placebo-controlled trial of topical steroid in presumed viral conjunctivitis. Br. J. Ophthalmol..

[92] Khan J., Mack H.G. (2020). Management of conjunctivitis during the COVID-19 pandemic 2020. Aust. J. Gen. Pract..

[93] Loffredo L., Pacella F., Pacella E., Tiscione G., Oliva A., Violi F. (2020). Conjunctivitis and COVID-19: A meta-analysis. J. Med. Virol..

[94] Chen L., Liu M., Zhang Z., Qiao K., Huang T., Chen M., Xin N., Huang Z., Liu L., Zhang G. (2020). Ocular manifestations of a hospitalised patient with confirmed 2019 novel coronavirus disease. Br. J. Ophthalmol..

[95] Solano D., Virgile J., Czyz C.N. (2020). Viral Conjunctivitis. StatPearls.

[96] Skevaki C.L., Galani I.E., Pararas M.V., Giannopoulou K.P., Tsakris A. (2011). Treatment of Viral Conjunctivitis with Antiviral Drugs. Drugs.

[97] Zhou Y., Zeng Y., Tong Y., Chen C. (2020). Ophthalmologic evidence against the interpersonal transmission of 2019 novel coronavirus through conjunctiva. medRxiv.

[98] Colavita F., Lapa D., Carletti F., Lalle E., Bordi L., Marsella P., Nicastri E., Bevilacqua N., Giancola M.L., Corpolongo A. (2020). SARS-CoV-2 Isolation From Ocular Secretions of a Patient With COVID-19 in Italy With Prolonged Viral RNA Detection. Ann. Intern. Med..

[99] Napoli P.E., Satta G.M., Coronella F., Fossarello M. (2014). Spectral-Domain Optical Coherence Tomography Study on Dynamic Changes of Human Tears After Instillation of Artificial Tears. Investig. Ophthalmol. Vis. Sci..

[100] Napoli P.E., Coronella F., Satta G.M., Fossarello M. (2014). A Novel Technique of Contrast-Enhanced Optical Coherence Tomography Imaging in Evaluation of Clearance of Lipids in Human Tears. PLoS ONE.

[101] Napoli P.E., Nioi M., Mangoni L., Gentile P., Braghiroli M., D’Aloja E., Fossarello M. (2020). Fourier-Domain OCT Imaging of the Ocular Surface and Tear Film Dynamics: A Review of the State of the Art and an Integrative Model of the Tear Behavior during the Inter-Blink Period and Visual Fixation. J. Clin. Med..

[102] Van Santvliet L., Ludwig A. (2004). Determinants of eye drop size. Surv. Ophthalmol..

[103] Hodos R.A., Kidd B.A., Shameer K., Readhead B.P., Dudley J.T., Khader S. (2016). In silico methods for drug repurposing and pharmacology. Wiley Interdiscip. Rev. Syst. Biol. Med..

[104] Xue H., Li J., Xie H., Wang Y. (2018). Review of Drug Repositioning Approaches and Resources. Int. J. Biol. Sci..

[105] Cohen J. (2020). Can an anti-HIV combination or other existing drugs outwit the new coronavirus?. Science.

[106] Wu K., Chen L., Peng G., Zhou W., Pennell C.A., Mansky L.M., Geraghty R.J., Li F. (2011). A Virus-Binding Hot Spot on Human Angiotensin-Converting Enzyme 2 Is Critical for Binding of Two Different Coronaviruses. J. Virol..

[107] Ito M., Baba M., Sato A., Pauwels R., De Clercq E., Shigeta S. (1987). Inhibitory effect of dextran sulfate and heparin on the replication of human immunodeficiency virus (HIV) in vitro. Antiviral Res..

[108] Kim S.Y., Jin W., Sood A., Montgomery D.W., Grant O.C., Fuster M.M., Fu L., Dordick J.S., Woods R.J., Zhang F. (2020). Characterization of heparin and severe acute respiratory syndrome-related coronavirus 2 (SARS-CoV-2) spike glycoprotein binding interactions. Antiviral Res..

[109] Schnitzler P., Schön K., Reichling J. (2001). Antiviral activity of Australian tea tree oil and eucalyptus oil against herpes simplex virus in cell culture. Die Pharm..

[110] Bishop C.D. (1995). Antiviral Activity of the Essential Oil of Melaleuca alternifolia (Maiden amp; Betche) Cheel (Tea Tree) Against Tobacco Mosaic Virus. J. Essent. Oil Res..

[111] Garozzo A., Timpanaro R., Stivala A., Bisignano G., Castro A. (2011). Activity of Melaleuca alternifolia (tea tree) oil on Influenza virus A/PR/8: Study on the mechanism of action. Antivir. Res..

